# Algorithmic Accountability in Context. Socio-Technical Perspectives on Structural Causal Models

**DOI:** 10.3389/fdata.2020.519957

**Published:** 2021-01-29

**Authors:** Nikolaus Poechhacker, Severin Kacianka

**Affiliations:** ^1^ Institute for Public Law and Political Science, University of Graz, Graz, Austria; ^2^ Department of Computer Science, Chair of Software and Systems Engineering, Technical University of Munich, Munich, Germany

**Keywords:** algorithms, structural causal model, pragmatism, accountability, causality, social theory

## Abstract

The increasing use of automated decision making (ADM) and machine learning sparked an ongoing discussion about algorithmic accountability. Within computer science, a new form of producing accountability has been discussed recently: causality as an expression of algorithmic accountability, formalized using structural causal models (SCMs). However, causality itself is a concept that needs further exploration. Therefore, in this contribution we confront ideas of SCMs with insights from social theory, more explicitly pragmatism, and argue that formal expressions of causality must always be seen in the context of the social system in which they are applied. This results in the formulation of further research questions and directions.

## Introduction

The rise of machine learning and automated decision making (ADM) affects many domains of social life. They have been used in court decisions (Angwin et al., 2016), policing (Kaufmann et al., 2019), hiring practices, and many more. Negative experiences with these systems led to a scholarly discussion in which formulations like *Weapons of Math Destruction* (O’Neil, 2016) or *Algorithms of Oppression* (Noble, 2018) have been used. As such, the power of algorithms and how to deal with these entities has become a major point of discussion (Ziewitz, 2016; Beer, 2017)—even creating its own field of *critical algorithm studies* (e.g., Gillespie, 2014; Seaver, 2018). Because of the intensifying application of these systems in various social domains, issues of fairness, (in)justice and power relations have become the focus of attention, especially in the form of bias (Friedman and Helen, 1996; Bozdag, 2013; Crawford, 2013). As a result, “algorithmic accountability” has been suggested as a means (e.g., Diakopoulos 2015) to mitigate the risks of bias and inequalities produced by algorithmic systems (Veale and Binns, 2017).

Accountability, however, is an ambiguous term in itself and was never clearly defined in computer science. Kacianka et al., (2017) found that most implementations of accountability do not use a peer reviewed definition of accountability and either provide no definition at all or rely on a loose dictionary definition. Despite being used as an umbrella term, accountability gained much prominence within the academic discussion, most prominently at the ACM Conference on Fairness, Transparency and Accountability. There, accountability is understood as “public accountability” and mostly follows the understanding of (Bovens, 2007, 9), who writes that “[t]he most concise description of accountability would be: “the obligation to explain and justify conduct”, although he also cautions that “[a]s a concept (...) “accountability” is rather elusive. It has become a hurrah-word, like “learning”, “responsibility”, or “solidarity”, to which no one can object” (Bovens, 2007, 9). This also seems to be true for algorithmic accountability. Wieringa (2020, 10). conducted a systematic literature review of the field of algorithmic accountability through the lens of Bovens. She found that the “term “algorithmic accountability” is inherently vague” and derives the following definition of algorithmic accountability, using the terminology and ideas presented by Bovens:

“Algorithmic accountability concerns a networked account for a socio-technical algorithmic system, following the various stages of the system’s lifecycle. In this accountability relationship, multiple actors (e.g. decision makers, developers, users) have the obligation to explain and justify their use, design, and/or decisions of/concerning the system and the subsequent effects of that conduct. As different kinds of actors are in play during the life of the system, they may be held to account by various types of fora (e.g. internal/external to the organization, formal/informal), either for particular aspects of the system (i.e. a modular account) or for the entirety of the system (i.e. an integral account). Such fora must be able to pose questions and pass judgment, after which one or several actors may face consequences. The relationship(s) between forum/fora and actor(s) departs from a particular perspective on accountability” (Wieringa, 2020, 10).

However, it is important to note that there is not just one definition of accountability. For example, in contrast to Bovens, Lindberg (2013), who is deeply critical of Bovens and the Utrecht school^1^, establishes accountability as a classical concept where subtypes are complete instances of their parents. In psychology, Hall et al. (2017) give a great overview of the concept of *felt* accountability, which focuses on the feeling of the individual. Besides *algorithmic* accountability, the state of the art on accountability in computer science is split into three branches of research.

First, works building on Weitzner et al., (2008) use the term “Information Accountability” to formulate a new approach to data control measures based on the idea of detection, rather than prevention. This approach does not try to prevent unauthorized data access, but wants to design systems in such a way that data access is logged and therefore any access to data is easily tracked. If data is “leaked”, it should be easy to identify the deviant entity and hold it accountable. Second, in the field of cryptographic protocols, Küsters et al. (2010) formalized accountability and linked it to verifiability. The main challenge here is to discover entities that attempt to falsify the results of elections. This requires a precise definition of a protocol, or allowed actions, to work. Recent advances in accountability for cryptographic protocols also started to investigate the use of causal reasoning for attributing blame (e.g. Künnemann et al. 2019). Third, accountability is discussed in the field of cloud computing, mainly focusing on data protection as well as accounting for resource usage (e.g. Ko et al. 2011).

The question remains how an algorithm can be hold accountable for its “actions”. Algorithms are often discussed in terms of opaque and powerful black boxes (Pasquale, 2015), which resulted in the often-formulated demand of algorithmic transparency. Yet it remains unclear how to implement algorithmic transparency and what its benefits would be (Ananny and Crawford, 2018). Accountability would require the translation of expert knowledge, such as algorithmic techniques, into accounts that are understandable to a broader audience. A task that is not easily achieved—especially when confronted with machine learning applications (Burrell, 2016; Neyland, 2016). Additionally, the ideal of transparency often collides with claims of intellectual property rights (Burrell, 2016). Thus, exploring alternative approaches of producing and thinking about accountability are needed.

In the recent debate, interpretability and explainability are discussed as alternatives to total transparency of algorithmic systems. Doshi-Velez et al. (2017) for example point out the importance of explanations to produce accountability. Instead of demanding absolute transparent systems, they argue that it suffices to know “how certain factors were used to come to the outcome in a specific situation” (Doshi-Velez et al., 2017, 7). This does not require full disclosure of the internal workings of an algorithmic system, but can be achieved by a statistical input/output analysis which results in a simplified model of human-readable rules explaining the observed data points. By this, the explanation system is an empirical reconstruction of the algorithm’s behavior. Such an explanation is not a one-to-one reconstruction of the internal workings, but an external model to find interpretable rules to explain the algorithm’s actions.

But not only in legal settings is causality important for explanations and achieving fairness (Madras et al., 2019). Mittelstadt et al. (2016) argue for the relevance of causality and causal knowledge in ethical considerations regarding AI and machine learning. Wachter et al. (2017a) extended this approach by suggesting counterfactuals. Counterfactuals are deviations from observed input data that are used to reconstruct relations between input and output that goes beyond the actual application. By this, explanations in form of differences in the input data that make a difference in the results can be reconstructed, e.g. using varying variables on race or gender to see if the results change (Mittelstadt et al., 2019). Further, Wachter et al. (2017b) argue that counterfactual explanations meet the legal requirements formulated under the GDPR. However, formulating potential influence of input data points on the behavior of agents requires a post-hoc explanation of causality (Miller, 2019; Mittelstadt et al., 2019). If we formulate rules describing the impact of input data on the classification of an algorithmic system, we are basically modelling a causal relationship to grasp observed behavior that goes beyond mere correlation. Mittelstadt et al. (2016) therefore argue for the relevance of causality and causal knowledge in ethical considerations regarding AI and machine learning. Madras et al. (2019) even argue that counterfactual causal models are able to produce fairer systems, as the influence of hidden confounding variables can be discovered. As a result, causality seems to be a promising approach to tackle issues of algorithmic accountability. This led computer science scholars to explore the formal expression of algorithmic accountability as a structural causal model (SCM) (Kacianka and Pretschner, 2018). The underlying idea is that accountability always requires causality (Kacianka et al., 2020). This approach extends the argument for causality as an essential feature beyond the notion of explainability. It assumes that a person cannot be held accountable for actions s/he did not cause, therefore referring to the social and political function of causality in human reasoning (see also Miller, 2019; Mittelstadt et al., 2019). In doing so, it also signifies the importance of the underlying models of causality and the process of their (social) construction.

SCMs (Pearl and Mackenzie, 2018) represent a human-readable graphical model, while also offering mathematical tools to analyze and reason over them. For example, take two definitions of accountability: One states that a system is accountable, if its logs might be reviewed by some third party. The second one defines an elaborate framework of checks and balances, in which every action of a computer system is reviewed by a human principal. Both are valid definitions of accountability and in line with recent literature. The first example is similar to the notion of felt accountability used in psychology (Hall et al., 2017), while the second one resembles the Responsible-Accountable-Consult-Inform (RACI) framework used in the organizational sciences (Smith et al., 2005). Both models can be expressed as a SCM and matched to a technical system. If a system takes an undesired action, its SCM will allow us to understand why this undesired action happened. If the SCM corresponds to an accountability definition, we can also see who is to be held accountable for the system’s undesired action.

Yet an open question is how accountability, expressed as SCMs, can take the social structure in which they are placed into account i.e. consider, which forms of accounting (Neyland, 2016) for their actions are compatible with the practices (re-)produced within the social domain. We therefore confront ideas of SCMs with insights from the social sciences and humanities and argue that formal expressions of causality must always be seen in the context of the social system in which they are applied. The argument operates with the observation that SCMs are being discussed as means for producing algorithmic accountability and situates this observation in an interdisciplinary perspective. In this contribution, we will first introduce how causality can be expressed in SCMs and then contrast the method with concepts from interactionist theories of social science, more specifically pragmatism, to theorize the interaction effects between causal models of algorithms and the social interaction order in which they are placed.

## From Causality to Accountability: The Computer Science Approach

While correlation does not imply causation is a well-known mantra, hardly anyone can give a mathematical formalization of causality. Recently, Pearl and Mackenzie (2018) put forward a formalization of causality that extends structural equation models to SCMs, but the expression of causality comes with its own methodological challenges. The gold standard of determining causality has been the randomized controlled trial (RCT), which has been popularized by RA Fisher (for a historical perspective see Pearl and Mackenzie, 2018, 139). In such experiments, the investigators try to create a stable environment and manipulate only a single variable (ceteris paribus). The fundamental downside of RCTs is that they are often infeasible, unethical or prohibitively expensive (Pearl and Mackenzie, 2018).

The alternatives to RCTs are observational studies, where researchers gather data and try to understand some (causal) processes. In these settings, researchers cannot directly manipulate any factors and are therefore restricted to recorded data, which makes the reconstruction of causal relations problematic. Pearl was the first to show that even with observational data causality could be proven (Pearl and Mackenzie, 2018). According to Pearl, SCMs allow expressing an understanding of a process’ causal structure, which can be formally expressed as follows:

Formally, an SCM is a tuple M = (U,V,F) where U is a set of exogenous variables, V is a set of endogenous variables, F associates with each variable in X ∈ V a function that determines the value of X given the value of all other variables.

It is noteworthy that the universe of discourse is split into two sets of variables: exogenous variables, which are taken as given and for which no explanation can be provided, and endogenous variables that are considered relevant for the causality relations. Additionally, a given understanding of the causal relation is modeled by a set of functions in F that describe the mathematical relation between the variables. By not having a causal relation between variables in such a model, it is assumed that one variable cannot influence another. In the graphical model corresponding to the SCM this is shown by the absence of arrows between two variables.

**FIGURE 1:**
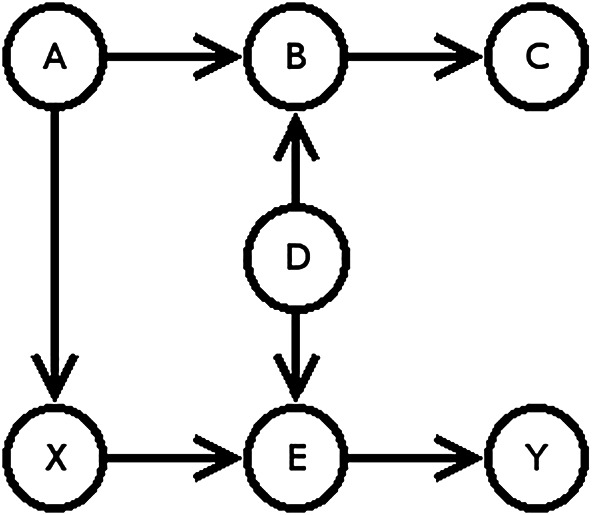
A simple causal model following the definitions of (Pearl and Mackenzie, 2018, 159) (created by the authors).

In Figure 1, one can mathematically express that B has no effect on Y, but that X is causally linked to Y. The caveat of this modeling approach is that any statement of causality depends on the underlying model. We, as experts, are forced to state our assumptions and invite others to challenge them. While there is no way to prove a causal model correct, we can use data to refute some. However, for any given model, we can most likely find an alternative model, that will also explain the data.

Drawing on forms of causal modeling and applying them on algorithmic systems provides us with the possibility to express accountability of algorithmic systems in mathematical terms. Once a notion of accountability has been agreed upon, it can be expressed as a SCM. This formalization determines what data needs to be collected, making it possible to design systems in a way that sensitive data, such as gender or race, will not need to be stored. In Figure 1, for example, if B was gender and we were only interested in Y, we can show that B does not affect Y and that we therefore do not need to record B.

**FIGURE 2:**
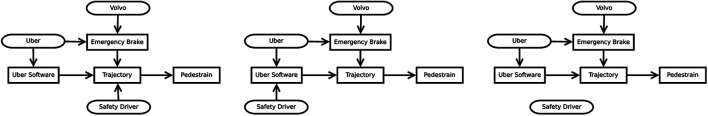
Three possible SCMs for the Uber case (from left to right) **(A)** The human can take over, **(B)** Human Influence is moderated by the machine, **(C)** No human influence is possible (created by the authors).

To illustrate our point, we will expand on the example given by Kacianka and Pretschner (2018). They examined a prominently reported accident involving an autonomous vehicle operated by Uber in Arizona (Elish, 2019). In this unfortunate accident, the test vehicle was driving autonomously and had a safety driver on board. However, the system mis-detected a pedestrian crossing the road, fatally injuring her. The safety driver on board of the car was distracted and did not manage to operate the brake in time, while the emergency braking system designed by the chassis manufacturer was disabled, because it interfered with the autonomous driving capabilities of the car. In the aftermath, one of the questions asked was who was to be held accountable for the accident. This example can now be modeled as an SCM (Kacianka et al., 2020). The answer to who is to be held accountable in the ultimately depends on the causal understanding of the events. The Figure below depicts three possible causal configurations:

Figure 2A shows a configuration in which the human has direct influence on the trajectory of the car. This influence is moderated by the software in Figure 2B, and in Figure 1C the human has no influence on the course of events at all. When talking about autonomous cars, many people will often have the causal model shown by Figure 2C in mind, while Figure 2B or even 2a are equally valid explanations. SCMs (Pearl and Mackenzie, 2018), as used in Figure 2, offer a mathematically precise way to express such causality relations. The arrows denote causal connections, while the boxes denote variables. Here, rectangular boxes represent components, and rounded boxes represent natural or juridical persons. Even if we do not specify the exact mathematical function for each relation, we can reason about some form of causality. For instance, the absence of an arrow in Figure 2C, between the *Safety Driver* and the *Trajectory*, expresses that there is no causal connection between them. On the other hand, in Figure 1C, we could specify the exact influence of the components on the *Trajectory*. Simplifying to a Boolean formula, where *false* means *do nothing* and *true* indicates a c*hange in trajectory*, the formula could be: *Trajectory = Brake or Software or Driver*

To model that the system only brakes if both the emergency brake and the system agree, we could model it like this: *Trajectory = (Brake and Software) or Driver*

Now, once we have such a causal representation of a system, we can start looking for patterns of accountability. Following Kacianka et al. (2020), we can express definitions of accountability as causal models. For example, they use the definition of (Lindberg, 2013, 209), who conceptualizes accountability as:

1. An agent or institution who is to give an account (A for agent);
2. An area, responsibilities, or domain subject to accountability (D for domain);
3. An agent or institution to whom A is to give account (P for principal);
4. The right of P to require A to inform and explain/justify decisions with regard to D; and
5. The right of P to sanction A if A fails to inform and/or explain/justify decisions with regard to D.

**FIGURE 3:**
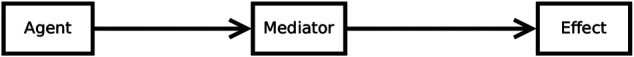
The causal model for the Lindberg accountability pattern; the principal is not part of the pattern. Taken from Kacianka et al. (2020).

This can be expressed as in the causal model shown in Figure 3

We can draw different conclusions about accountability from the graphical representations of causality. Coming back to the different models of causality in Figure 2, we are confronted with different possibilities. We can see that in Figure 2A, the software, the emergency brake, and the safety driver, are accountable for the accident. However, looking at Figure 2C, we can see that the safety driver is no longer connected to the pattern and thus cannot be held accountable. We want to emphasize that we do not argue for the correctness of the specific models. Both the model of the system, as well as the model of accountability, can be improved, changed and refined. Rather we want to show that the usefulness of SCMs is that they allow us to express these causal relationships and offer us a formalization to clearly state our assumptions. These assumptions can then be discussed and criticized, and joint models can be developed.

Though, taking up the warning of Mittelstadt et al. (2019), models should be used carefully. In the Uber example, the different definitions of accountability provided will result in different understandings: one could conclude that Uber was accountable or that the driver was accountable. While none of the two definitions is inherently wrong on a formal level, a shared definition of accountability is needed to resolve the issue. This insight on the contingency in modeling leads to the conclusion that models of causality are so-called second order observations. They formalize not only how a causal relation can be expressed, but they also have to consider how the social system experiences causalities and creates spoken or written accounts about them. As such, accountability of algorithmic systems is not merely about making actions of an algorithm understandable through causal reasoning, but should also address the question of towards whom such an algorithmic system should be accountable (Neyland, 2016). In other words, the algorithmic system must be accountable to the principals with whom it interacts in the given situation. This implies two conditions. First, the causal model for the algorithmic system needs to be aligned with the actual application in the specific social system in which it is placed. Second, the model and its assumptions must not only be accountable to the developers of the model, but also the accounts created by a SCM have to be interpretable by the other members of the social system. Briefly: the SCM must be seen in context.

## Putting Structural Causal Models Into Context

Causality as a concept has been discussed in the social sciences for a long time. Thus, contrasting and confronting the notion of causality from the formal perspective of computer science with the approach of pragmatism, could produce insights into the social interfaces between algorithmic accountability and social structure. As described before, different SCMs can be applied to explain given data. The question then is, which models correspond with shared expectations and practical enactments of accountability, and to which extent. Coming back to the Uber example, the model could describe both the missing reaction of the driver or the action of the board-computer not applying the brakes as a causal factor for the accident. Formally, both factors might explain the accident, but they relate to different normative assumptions.

The multiplicity of possible explanations is thereby not unique to causal modelling in computer science and neighboring fields, but touches upon a general epistemic position and how the world is experienced by individuals and communities. An influential tradition that deals with questions of social construction of truth and collective expectations has been American pragmatism (James, 1907 2014; Thomas and Thomas, 1928). American pragmatism was foremost a philosophy developed in the U.S. at the beginning of the 20th century. Later it became an influential way of thinking within the social sciences, exemplified by the Chicago School of Sociology (e.g., Park, 1915) and Symbolic Interactionism (Blumer, 1986; Dewey, [1925] 2013). Besides, it has been understood by its scholars as an empirical philosophy (see Dewey, [1925] 2013), which conveys an interesting branch of thinking that focuses on the practices and interactions of individuals and how bigger patterns and social worlds emerge from them (Strauss, 1978; Bowker and Star, 2000).

Two conceptions of human action are of importance when discussing causality as a mode of accountability production. First, pragmatists argue that human action is tied to problem solving. What we do, and what results out of our actions, is tied to the perception of a problem that needs to be solved, and our positioning in the world. These problems that need to solved are not objectively given, but are experienced and imagined as such by an individual or a group of individuals (Marres, 2007). Thus, firstly, when looking at patterns of communication or interaction, the question arises what problem and—more importantly—whose problem is solved by the observed behavior. Secondly, the perception of the world cannot be separated from these problem-solving activities. What is true or real is experienced in our practices, in testing and updating our assumptions about the world. Truth therefore becomes a question of practicability and “what works” (James, [1907] 2014). Nevertheless, in a given situation different perceptions and imaginations of truth can work in the practical doing. As a result, sharing a common vision of the world is not necessarily given, but must actively be produced through processes of socialization (Thomas and Thomas, 1928). This contingency of perceived reality must then be considered when talking about causality.

For Dewey—an important pragmatist scholar at the beginning of the 20th century—causality represents a sequential order of events, though he doesn’t see causality as the result of pre-existing associations between these events (Dewey, [1938] 2007). Instead, the associations between these events are operational, i.e. associations are constructed in a social process in order to solve a given problem of inquiry. In this perspective, the notion of causality is insofar problematic, as the assumption that an event A caused event B is in itself a reduction within an endlessly more complex situation. For each event A that we can identify, we can also identify more events that caused it, moving to ever finer-grained levels of interactions. Meanwhile, event B is not necessarily the end of a potential endless chain of causations (see also Stone, 1994).

Taking our example of the Uber-car accident, we could now ask for the initial event. Was it the driver, who was braking too late? The system mis-classifying the pedestrian? The driver starting the car, or maybe even the engineers, who assembled the system? All of these events would be viable starting points for a chain of causality. Similarly, we could argue that it wasn’t the hit of the car, which killed the pedestrian, but that the hit damaged some inner organs, which led to internal bleeding, which then led to insufficient oxygen supply to the brain, etc.^2^ Reducing this complex process to a relation between e.g. the classification and the pedestrian’s death represents a simplification, which Dewey termed “common sense causation” (Dewey, [1938] 2007). This also includes questions of co-correlations of events, which could lead to different common-sense causations. As such, the model of causality needs a link to learned experiences of the social system’s members to create *plausible* accounts on their own actions (Dewey, [1925] 2013; James, [1907] 2014). Similar concepts can be found in cognitive psychology (e.g., Pylyshyn, 2006). Causality therefore not only describes the associations between different identifiable events, but also presumes a shared construction of the world.

There is an interesting convergence of arguments between social theory, the formulation of structural causal models, and algorithmic accountability. In the context of algorithmic accountability, Mittelstadt et al. (2019) already argue that explanations of algorithmic behavior should resemble the recipient’s epistemic and normative values. In terms of social theory, this now addresses the constructions of a social system. Further the “common sense causation”, as described by Dewey, has been introduced in SCMs as “context setting” (Halpern, 2016). Context setting defines the elements to identify and include in the model auf causal relations. This, however, can be seen as a specific setting of how reality is being perceived and imagined within SCMs. During the modelling of these SCMs, specific ideas and assumptions about reality are being inscribed into these models.

Pearl and Mackenzie (2018) base their model of causation on a question that is not too different to a pragmatist conception of causality. They follow a definition of causal reasoning that is not asking for the essence of causality, or, to put it differently—for an objective, neutral, and detached definition of such a term—but for the performatively produced understanding of causality. The explanatory power of SCMs is granted, as “causal inference is objective in one critically important sense: once two people agree on their assumptions, it provides a 100 percent objective way of interpreting any new evidence (or data)” (Pearl and Mackenzie, 2018, 91). This means that if several models describe reality in a way that is functional for a given problem definition, objectivity is achieved through the act of commonly deciding on which model is the most useful. This has important implications on how SCMs can be applied within a social system to produce accountability.

Arguing for the possibility of multiple models of causal relationships that have to be negotiated means to assume the (important) position of an external observer. In order to become objective in the terms of Pearl and Mackenzie, the reasoning over different possible causal models have to be aligned with the interpretation of other observers. The question then is not only if a model converges with the perceived reality of the developers, but instead observations of a second order are necessary, to produce models that are also plausible to these other observers. This raises the question how causality is being described within a social system in which the model should be deployed, i.e. observing how the social setting is observing reality.^3^ Causal models rely on a shared understanding of the world and a common form of causal reasoning. What Pearl and Mackenzie are implicitly referring to has been conceptualized in pragmatism as shared knowledge and the production of intersubjectivity (Mead, 1917 and Mead, 1967). Acting and reasoning are based on experiences that create implicit causal models of the world. The interpretation of newly gathered data—here seen as a new experience—can only be interpreted according to the experiences one has made in the past. This therefore requires a deep understanding of the social interaction system in which causal models should operate.

Coming back to our example of the Uber accident, the question arises, which of the presented models coheres with society’s perceptions. For the causal description of the Uber case, the question therefore is not which SCM is better, but which ones best reflects the normative and experienced causalities of the social groups, for which it should solve the problem. This of course entails interests of different social groups and therefore requires a broader discussion among them. The model displayed in Figure 2C might seem intuitive to many people, as the term “autonomous driving” suggests that the car is acting “on its own”, and thus the company who built the car should be held accountable, while insurance companies and producers of autonomous cars would probably prefer the models described in Figures 2A or 2B. When it comes to legal decisions and settings, one has to not only attribute accountability, but also responsibility.

Each of the models displayed represents a valid reduction of a highly complex reality into a manageable set of entities and relations. However, the normative ideas and social consequences of these models differ to a large degree. Thus, for SCMs to be able to act as *accountability machines*, they have to reflect these social constraints in order to become objective. *Accountability* then does not (only) mean to produce addressable entities that can be held responsible, but to create a model that is able to give account about what happened in a way that is understandable and acceptable to the members of the addressed social group. Constructing causal models therefore requires knowledge about these different modes of attributing and producing accountability within different functional interaction systems.

## Conclusion and Outlook

Causality and the calculation of counter-factuals is a promising approach to algorithmic accountability. By calculating and formulating human-readable rules to explain the observed behavior of an algorithmic system, they can be made available to public scrutiny. Especially, as an implementation of causal descriptions can be applied in a way that balances the public’s need to know and the protection of intellectual property rights. Thus, the introduction of such *explainability systems* (Doshi-Velez et al., 2017) creates an interface between the practices of the developers of algorithmic systems and the organizations and communities that want or need to hold them accountable. This could create the means to intervene in the production and deployment of algorithmic systems.^4^ Producing *account*-ability, in terms of being able to understand and interpret the behavior of algorithms, also creates *contest*-ability, i.e. the ability to reject specific implementations. However, in building accountability systems, we have to be aware of the construction of causality within the social system in which these machineries should be able to operate. That is, in order to enable a community to hold algorithms and their developers accountable, SCMs operate as an interface between the practices of designers and the practices of the social system’s members.

The Uber case illustrates that different models of causality correspond with social imaginaries of common-sense causality to varying degrees. The legitimacy of SCMs as accountability machines therefore hinges on the relation between these different conceptions of causality. If social research and computer science are to collaborate in the development of SCMs as a means to produce accountability, more, and especially interdisciplinary research on the matter is necessary. It remains an open question, how translating social visions of fairness, discrimination, and “normality” into mathematical models can be achieved in a way that enables interactions between algorithms and their social context. This calls for more in-depth studies of interaction patterns between algorithms, social systems, and SCMs as translation devices between the technical and social realm. Such studies would enable the development of SCMs, that could express causalities in a field’s *own language*. Simultaneously, it would be naïve to assume that there was only one existing construction of accountability between different institutions and actors. By making these different notions of accountability visible, SCMs can therefore not only foster disagreement with single observers, but with different communities, each with their own normative account and resulting constructions of causality.

Opening up the discussion about possible causal models could therefore also be a means to a broader and (deliberative) democratic discussion about how algorithms should operate within our societies. Instead of treating algorithms or causal models as given, the perspective explored here calls for more inclusive forms of development, as algorithms and statistical models are not objective by nature. Bringing developments of algorithmic systems and SCMs (as accountability machines) into conversation with the normative ideas and imaginaries of the social system within which they are operating, could therefore not only result in *account*-able, but also more responsible systems. The question, if and how algorithmic accountability can foster social integration, therefore needs further inquiry.

This leads us to three major questions for future research: First, how do people actually make meaning of their everyday life in relation with algorithms, especially in different (public) organizations? Second, how can these processes of meaning-making be translated into SCMs in a way that is compatible with the social system? And third, how can these models consider that different modes of constructing accountability and causality are being negotiated in social systems? These questions call for a closer cooperation between several disciplines, including philosophy (of technology), legal studies, ethics, social science and computer science, to name just a few. Ethnographic studies of algorithmic systems in action and quasi-experimental studies would be a valuable contribution to the technical implementation of SCMs with interdisciplinary research. It therefore seems productive to explore the possibilities of constructing algorithmic accountability by bringing perspectives and interdisciplinary approaches into an ongoing conversation with SCMs.

## Author Contributions

Both authors, NP (lead author) and SK (co-author) made substantial contributions to the conception, drafting and finalizing of the theoretical analysis work, whereas NP provided social theory insights and the synthesis of causal modelling and social theory, whereas SK was responsible for description of SCMs. The text is a joint effort, therefore it is not possible to attribute sections to one author alone.

## Funding

The authors acknowledge the financial support for open access publishing by the University of Graz. Severin Kacianka's work was supported by the Deutsche Forschungsgemeinschaft (DFG) under grant no. PR1266/3-1, Design Paradigms for Societal-Scale Cyber-Physical Systems.

## Conflict of Interest

The authors declare that the research was conducted in the absence of any commercial or financial relationships that could be construed as a potential conflict of interest.

## Footnotes

1 Lindberg (2013, 203) writes that “[t]he main achievement [of Bovens and the Utrech School] is to obfuscate the distinctiveness of accountability from other types of constraints on actors’ power to act autonomously. When the term “sanction” finally is misunderstood to denote only punishment (deviating from the proper meaning of the word in English), the paraphrasing becomes misleading.”

2 We would like to note that this is a hypothetical reflection of this unfortunate event and that we did not inquiry into the exact medical circumstances.

3 There is of course a long-standing discussion about these issues in (the history of) cybernetics and social system theories (e.g. Luhmann, 1996; Hayles, 2012).

4 This can become even more important if we think about the complicated relationship between innovation and law, and the way how innovations like autonomous driving can be made accessible to legal reasoning and regulation (Eisenberger, 2016).
